# Adolescent with Diabetic Ketoacidosis, Hypothermia and Pneumomediastinum

**DOI:** 10.21980/J8FP8J

**Published:** 2023-10-31

**Authors:** Steven Millner, Courtney Devlin

**Affiliations:** * University of Pittsburgh Medical Center Harrisburg, Department of Emergency Medicine, Harrisburg, PA

## Abstract

**Audience:**

The target audience of this simulation is emergency medicine residents and medical students. The simulation is based on a real case of a 12-year-old male who presented obtunded with shortness of breath and hypothermia who was ultimately diagnosed with diabetic ketoacidosis (DKA) and pneumomediastinum. This case highlights the diagnosis and management of an adolescent with new onset diabetic ketoacidosis and pneumomediastinum with deterioration of status, as well as important ventilator settings if intubation is required in the setting of diabetic ketoacidosis.

**Background:**

Type 1 diabetes is a common disease in the pediatric population with the prevalence being approximately 2.15 per 1000 youths and diabetic ketoacidosis being the presenting status in 30–40% of the patients.^1^ Physicians who evaluate a child with altered mental status must have diabetic ketoacidosis in their differential. In the setting of mechanical ventilation in patients with diabetic ketoacidosis (DKA), special care must be taken. Mechanical ventilation in these patients comes with increased risk, morbidity, and mortality. Risk factors for pneumomediastinum include lung disease such as asthma, chronic obstructive pulmonary disease (COPD), and malignancy, but also can occur in the acute setting of vomiting or trauma.^2^

**Educational Objectives:**

By the end of the simulation, learners will be able to: 1) develop a differential diagnosis for an adolescent who presents obtunded with shortness of breath; 2) discuss the management of diabetic ketoacidosis; 3) discuss management of hypothermia in a pediatric patient; 4) discuss appropriate ventilator settings in a patient with diabetic ketoacidosis; and 5) demonstrate interpersonal communication with family, nursing, and consultants during high stress situations.

**Educational Methods:**

This is a high-fidelity simulation that allows learners to manage the diagnosis and treatment of diabetic ketoacidosis and hypothermia in an adolescent patient. Participants participated in a debriefing after the simulation. There should be approximately 4–5 learners per case. This simulation was performed in 3 sessions. Each learner performed this simulation one time.

**Research Methods:**

The effectiveness of this case was evaluated by surveys given to learners after debriefing. Learners gave quantitative and qualitative results of their feedback using a 1–5 rating scale and open-ended written questions. This case was trialed with residents in their first through third years of training as well as fourth year medical students.

**Results:**

Feedback was very positive, with 19 residents completing the post-simulation survey. They enjoyed the case and reported they would feel more comfortable in a comparable situation in the future. Four survey questions were asked of the participants. On average, learners stated they felt the simulation improved their ability to manage a pediatric DKA patient, and their knowledge of complications and appropriate ventilator settings improved (modes of 5, 4 and 5, respectively).

**Discussion:**

Diabetic ketoacidosis is a common and critical diagnosis for emergency medicine physicians to consider in the setting of altered mental status in a pediatric patient. This simulation has multiple steps and is based on a real case of an obtunded and hypothermic pediatric patient who was ultimately diagnosed with diabetic ketoacidosis complicated by pneumomediastinum.

**Topics:**

Diabetic ketoacidosis, pneumomediastinum, hypothermia, altered mental status, pediatrics, adolescent, intubation, hypoxia, ventilator settings, cardiac arrest, emergency medicine, medical simulation.

## USER GUIDE

[Table t1-jetem-8-4-s1]

**Table t1-jetem-8-4-s1:** 

List of Resources:	
Abstract	34
User Guide	36
Instructor Materials	38
Operator Materials	48
Debriefing and Evaluation Pearls	50
Simulation Assessment	54

**Learner Audience:**

Medical Students, Interns, Junior Residents, Senior Residents

**Time Required for Implementation:**

**Instructor Preparation:** 20 minutes

**Time for case:** 20 minutes

**Time for debriefing:** 20 minutes

**Recommended Number of Learners per Instructor:** 4

**Topics:**

Diabetic ketoacidosis, pneumomediastinum, hypothermia, altered mental status, pediatrics, adolescent, intubation, hypoxia, ventilator settings, cardiac arrest, emergency medicine, medical simulation.

**Objectives:**

By the end of this simulation, the learner will be able to:

1. Develop a differential diagnosis for an adolescent who presents obtunded with shortness of breath
2. Discuss the management of diabetic ketoacidosis
3. Discuss the management of hypothermia
4. Discuss appropriate ventilator settings in a patient with diabetic ketoacidosis where learners appropriately match the patient’s minute ventilation
5. Demonstrate interpersonal communication with family, nursing, and consultants during high stress situations

### Linked objectives and methods

An obtunded adolescent patient presenting to the emergency department must be evaluated and treated quickly. The patient in this case is obtunded and hypothermic on arrival and quickly becomes unresponsive and hypoxic. Learners will be presented with a case of a critically ill pediatric patient who will require timely diagnosis and resuscitation. They will need to formulate an appropriate differential diagnosis (objective 1). The laboratory testing will reveal a blood sugar that reads “high” with associated acidosis, and chest x-ray will show pneumomediastinum. The learners will then initiate treatment of diabetic ketoacidosis (objective 2). Learners will have to administer IV fluids and insulin to treat the patient. Despite this, the patient will become hypoxic secondary to the patient’s pneumomediastinum requiring intubation, and the learners will be required to initiate appropriate ventilator settings for a patient with diabetic ketoacidosis. Given this patient is Kussmaul breathing at a rate of 30 breaths per minute, this rate must be matched in order to ensure the patient does not have worsening acidemia. Tidal volume may be calculated via approximately 6ml/kg of Ideal body weight (objective 3). Should inappropriate ventilator settings be requested, the patient will deteriorate to cardiac arrest requiring appropriate Advanced Cardiovascular Life Support (ACLS) for resuscitation. During the simulation, a family member will be present who will require frequent updates, and the learners should be able to communicate with them as well as nursing staff and the pediatric intensivist over the phone (Objective 4). At the end of the simulation, the learners will debrief and discuss appropriate management for patients they may encounter with similar presentations.

### Recommended pre-reading for instructor

1. Lodeserto F. Simplifying mechanical ventilation - part 3: Severe metabolic acidosis. REBEL EM - Emergency Medicine Blog. June 29, 2020. Accessed August 29, 2023. At: https://rebelem.com/simplifying-mechanical-ventilation-part-3-severe-metabolic-acidosis/
2. Nyce A, Byrne R, Lubkin CL, Chansky ME. Diabetic Ketoacidosis. In: Tintinalli JE, Ma O, Yealy DM, et al, eds. *Tintinalli's Emergency Medicine: A Comprehensive Study Guide, 9*^*th*^ *ed*. McGraw Hill; 2020. Accessed August 29, 2023. At: https://accessemergencymedicine.mhmedical.com/Content.aspx?bookid=2353&sectionid=190079125
3. Gripp KE , Trottier ED, Thakore S, Sniderman J, Lawrence S. Current recommendations for management of paediatric diabetic ketoacidosis. *Paediatr Child Health.* 2023;28(2):128–32. Posted December 5, 2022. Accessed August 29, 2023. At: https://cps.ca/en/documents/position/currentrecommendations-for-management-of-paediatric-diabetic-ketoacidosis

### Results and tips for successful implementation

The pilot session of this simulation was performed with emergency medicine residents in their first, second, and third years of training as well as fourth-year medical students. There were 4–5 learners in each simulation. We had a mixture of first- , second-, and third-year residents as well as medical students in all groups so there was no variation in complexity. There was a single nurse who was one of our education nurses. There was also a simulation technician who controlled our high-fidelity mannequin. There was also a family member who was portrayed by one of our education attending physicians who provided history. In this simulation, one tip would be to have clear instructions for the simulation technician for when to have the patient lose pulses. We were not clear enough with our technician, and when inappropriate ventilator settings were placed, the patient maintained pulses. The effectiveness of this simulation was measured using a survey rating the learners’ understanding of management of pediatric diabetic ketoacidosis. A scale of 1–5 where 1 (completely disagree) to 5 (completely agree) was used to answer four questions:

1. My knowledge of managing a pediatric patient with diabetic ketoacidosis was at my proper level of Post Graduate Year (PGY) training.
2. My knowledge after the simulation lab made me more prepared to manage a pediatric patient suffering from diabetic ketoacidosis requiring intubation
3. My knowledge of complications of DKA has increased due to this simulation
4. After completing this simulation lab, I will better recognize appropriate ventilator settings for a patient intubated with diabetic ketoacidosis.[Table t2-jetem-8-4-s1]

**Table t2-jetem-8-4-s1:** 

Question Number	1	2	3	4
Mode Response	4	5	4	5

In total, 19 residents and medical students completed the survey. All surveys were anonymous. The mode response for question 1 was 4, suggesting that participants had a good level of background knowledge of this disease process. For questions 2–4, which assessed how these participants felt about their knowledge after the case, the mode responses were 5, 4, and 5 respectively. They generally felt that this simulation enhanced their learning and understanding of how to treat a critical patient with diabetic ketoacidosis.

## Supplementary Information



## Figures and Tables

**Table t3-jetem-8-4-s1:** 

#1	Fingerstick glucose
#2	Chest radiograph
#3	Electrocardiogram
#4	Basic metabolic panel
#5	Complete blood count
#6	Venous blood gas
#7	Beta-hydroxybutyrate

**Table t4-jetem-8-4-s1:** 

**Vitals:**	Heart Rate (HR) 109	Blood Pressure (BP) 118/60
	Respiratory Rate (RR) 30	Temperature (T) 34.2°C
	Oxygen Saturation (O_2_Sat) 86% on room air	

**Table t5-jetem-8-4-s1:** 

*Fingerstick glucose*	HIGH

**Figure f1-jetem-8-4-s1:**
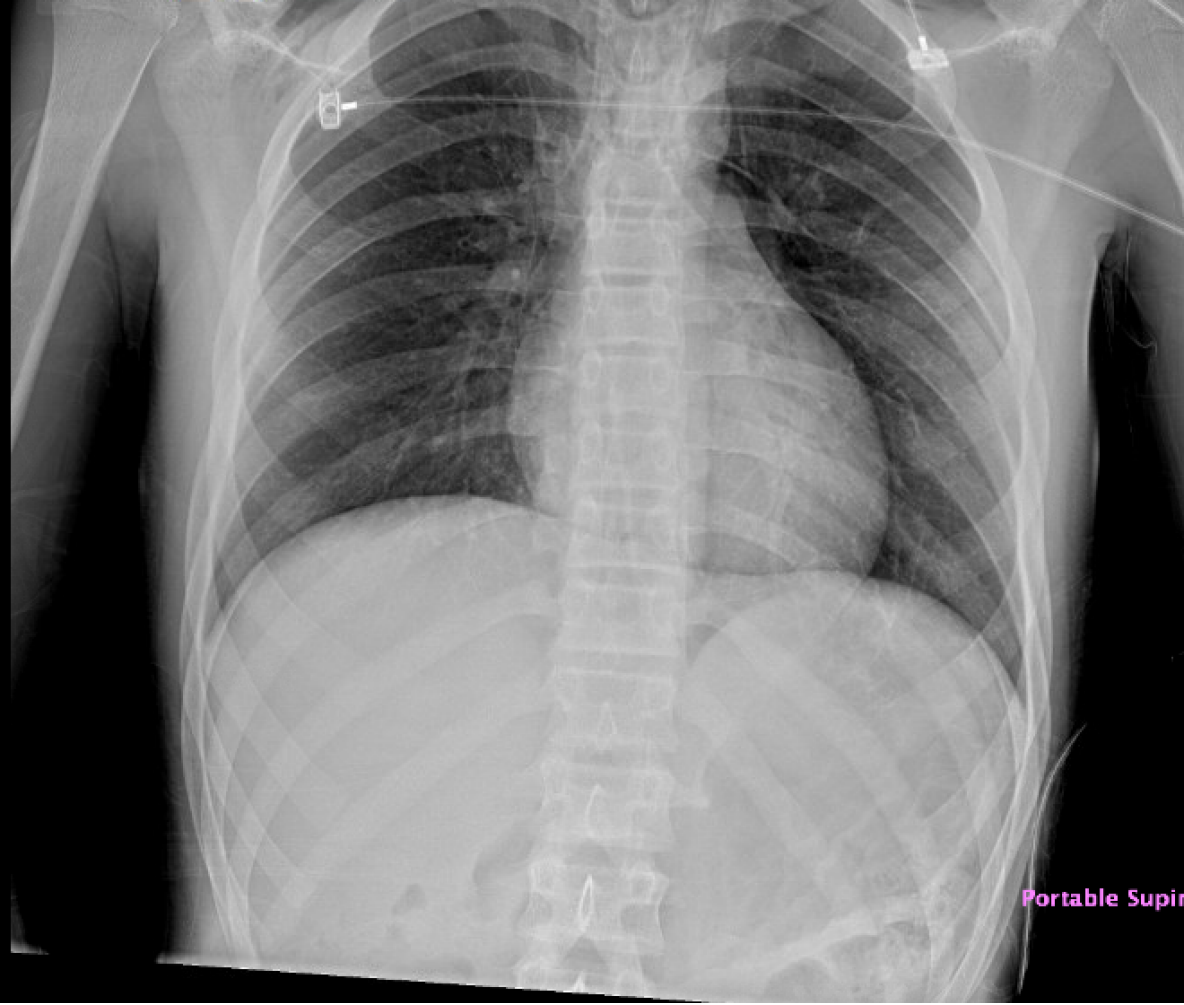


**Figure f2-jetem-8-4-s1:**
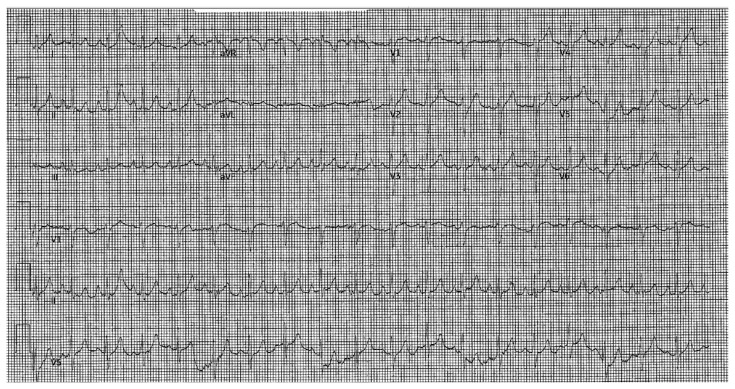


**Table t6-jetem-8-4-s1:** 

*Basic metabolic panel (BMP)*	
Sodium	128 mEq/L
Potassium	6.7 mEq/L
Chloride	90 mEq/L
Bicarbonate (HCO_3_)	3 mEq/L
Blood Urea Nitrogen (BUN)	34 mg/dL
Creatinine (Cr)	2.38 mg/dL
Glucose	>960 mg/dL
Calcium	10.9 mg/dL
Anion Gap	35.00 mmol/L

**Table t7-jetem-8-4-s1:** 

*Complete blood count (CBC)*	
White blood count (WBC)	39.2 × 1000/mm^3^
Hemoglobin (Hgb)	17.1 g/dL
Hematocrit (HCT)	56.3%
Platelet (Plt)	302 × 1000/mm^3^
RBC Count	6.77 ×1000/mm^3^
Neutrophils	86.0%
Lymphocytes	6.0%

**Table t8-jetem-8-4-s1:** 

*Venous blood gas (VBG)*	
pH	6.83
pCO2	20 mmHg
pO2	37 mmHg
HCO3	3 mEq/L
Venous	O2 Sat 36%

**Table t9-jetem-8-4-s1:** 

*Beta-Hydroxybutyrate*	>13.5 mmol/L

**Table t10-jetem-8-4-s1:** SIMULATION EVENTS TABLE:

Minute (state)	Participant action/trigger	Patient status (simulator response) & operator prompts	Monitor display (vital signs)

0:00 (Baseline) Stage 1	Initial Assessment	Patient obtunded and moaning.	T Not given HR 109 BP 118/60 RR 30 O2 86%

	Place on oxygen via non-rebreather	Patient oxygen improves to 100% on non-rebreather with no change in mental status. If patient is placed on nasal cannula, patient should not saturate above 90%. If no supplemental Oxygen administered, patient should decline to SPO2 80%.	Non-rebreather: HR 109 BP 118/60 RR 30 O2 100% NC: O2 90% No oxygen: O2 80%

	Ask for temperature	Nurse reports temperature is 34.8°C rectally. If this is not asked for, nurse should prompt by saying, “The patient feels cold.”	

	Patient placed on Bair hugger or warm blanket	If no action for hypothermia, nurse should ask if this temperature is acceptable.	Should the temp improve? Please provide what updated vitals should be.

2:00	Obtain history from family	Grandparents should mention patient seemed anxious and short of breath so they gave the patient lorazepam this morning. Has been vomiting for the past two days.	

	Obtain IV Access		T HR 114 BP 118/64 RR 32 O2 Wt 56kg

	Hyperglycemia	BGM reads “High.” IV fluids should be ordered. Patient should be given a 10–20mL/kg normal saline or Lactated Ringers fluid bolus, and then commence with a 1.5x maintenance fluid rate, which in this patient should be 144 ml/hr.	T 34.8° HR 114 BP 118/64 RR 32 O2 98%
		If participants fail to check BGM, patient should increase RR to 40, and hypoxia will progress. If learners continue to fail to ask for BGM, nurse should prompt by asking if learners would like a blood sugar. If participants fail to start IV fluids, HR should continue to climb to 120 BPM.	T 34.8° C HR 120 BP 118/64 RR 40 O2 98%

5:00 Start of Stage 2	Hypoxia	After IV fluids are started, SP02 should fall to 75%.	T 35.5°C HR 110 BP 118/64 RR 32 O2 75%

	Participants intubate patient	When participants intubate patient SPO2 should be 100%. Rapid sequence intubation should be performed with adequate paralytic and sedation such as Ketamine 1–2mg/kg, Etomidate .2–.4 mg/kg or Propofol 1– 2mg/kg for sedation with either Rocuronium 1.2mg/kg or Succinylcholine 1.5 mg/kg. If participants fail to intubate patient, SPO2 should continually decline until 0% O2 and patient should go into PEA arrest.	BP 118/60 HR 110 RR(per ventilator) SPO2 100%

10:00 Start of Stage 3	Respiratory rate on ventilator set	Ventilator should be set at a respiratory rate of at least 27 breaths per minute. If learner does not immediately recommend settings, confederate playing respiratory therapist should ask “what ventilator settings would you like?” If respiratory rate set at less than 27 breaths per minute, patient should undergo PEA arrest (10:00A).	This step can be skipped for Medical Students or PGY-1s

12:00A	PEA arrest	If PEA arrest is initiated, participants should recognize that profound acidemia is the cause and participants should give bicarbonate. If learners do not realize their error, nurse or respiratory therapist can ask, “do you think there was a problem with the ventilator settings?” or other cue to assist learners.	This step can be skipped for medical students or PGY-1s

12:00B	Order diagnostic studies	If iSTAT is ordered, provide BMP and VBG results. X-ray chest shows proper placement of endotracheal tube and pneumomediastinum. If participants order bedside ultrasound, verbalize normal echocardiogram. If ECG is obtained, it shows sinus tachycardia.	

15:00	Blood work results available	With review of the lab results, an insulin drip should be started. Participants should be allowed to otherwise treat for hyperkalemia as they choose. Supplemental potassium of 40mmol/L should not be administered secondary to hyperkalemia. If participants try to give potassium, they should be prompted by the nurse that the potassium was 6.7.	

	Management of pneumomediastinum	Participants should recognize pneumomediastinum needs no further management or consult thoracic surgery for input.	

17:00	Disposition	Call PICU to arrange admission. If surgery is called, they will recommend PICU admission and will consult on patient with no further recommendations at this time. If participants fail to call PICU, pediatric hospitalist should decline the patient.	

20:00 (Case Completion)	Simulation Ends		

**Table t11-jetem-8-4-s1:** 

**Critical Actions:** **Stage 1 Actions:** Vital signs Obtain history from grandparent Discuss warming Check blood sugar Administer IV fluids **Stage 2 Actions** Intubate patient Input correct ventilator settings If patient undergoes cardiac arrest from improper ventilator settings, discuss cause Start insulin and discuss IV fluids based on lab results Recognize pneumomediastinum Admit to PICU	0:00

**Table t12-jetem-8-4-s1:** 

	Milestone	Did not achieve level 1	Level 1	Level 2	Level 3
1	**Emergency Stabilization (PC1)**	□ Did not achieve Level 1	□ Recognizes abnormal vital signs	□ Recognizes an unstable patient, requiring intervention Performs primary assessment Discerns data to formulate a diagnostic impression/plan	□ Manages and prioritizes critical actions in a critically ill patient Reassesses after implementing a stabilizing intervention
2	**Performance of focused history and physical (PC2)**	□ Did not achieve Level 1	□ Performs a reliable, comprehensive history and physical exam	□ Performs and communicates a focused history and physical exam based on chief complaint and urgent issues	□ Prioritizes essential components of history and physical exam given dynamic circumstances
3	**Diagnostic studies (PC3)**	□ Did not achieve Level 1	□ Determines the necessity of diagnostic studies	□ Orders appropriate diagnostic studies. Performs appropriate bedside diagnostic studies/procedures	□ Prioritizes essential testing Interprets results of diagnostic studies Reviews risks, benefits, contraindications, and alternatives to a diagnostic study or procedure
4	**Diagnosis (PC4)**	□ Did not achieve Level 1	□ Considers a list of potential diagnoses	□ Considers an appropriate list of potential diagnosis May or may not make correct diagnosis	□ Makes the appropriate diagnosis Considers other potential diagnoses, avoiding premature closure
5	**Pharmacotherapy (PC5)**	□ Did not achieve Level 1	□ Asks patient for drug allergies	□ Selects an medication for therapeutic intervention, consider potential adverse effects	□ Selects the most appropriate medication and understands mechanism of action, effect, and potential side effects Considers and recognizes drug-drug interactions
6	**Observation and reassessment (PC6)**	□ Did not achieve Level 1	□ Reevaluates patient at least one time during case	□ Reevaluates patient after most therapeutic interventions	□ Consistently evaluates the effectiveness of therapies at appropriate intervals
7	**Disposition (PC7)**	□ Did not achieve Level 1	□ Appropriately selects whether to admit or discharge the patient	□ Appropriately selects whether to admit or discharge Involves the expertise of some of the appropriate specialists	□ Educates the patient appropriately about their disposition Assigns patient to an appropriate level of care (ICU/Tele/Floor) Involves expertise of all appropriate specialists
9	**General Approach to Procedures (PC9)**	□ Did not achieve Level 1	□ Identifies pertinent anatomy and physiology for a procedure Uses appropriate Universal Precautions	□ Obtains informed consent Knows indications, contraindications, anatomic landmarks, equipment, anesthetic and procedural technique, and potential complications for common ED procedures	□ Determines a back-up strategy if initial attempts are unsuccessful Correctly interprets results of diagnostic procedure
20	**Professional Values (PROF1)**	□ Did not achieve Level 1	□ Demonstrates caring, honest behavior	□ Exhibits compassion, respect, sensitivity and responsiveness	□ Develops alternative care plans when patients’ personal beliefs and decisions preclude standard care
22	**Patient centered communication (ICS1)**	□ Did not achieve level 1	□ Establishes rapport and demonstrates empathy to patient (and family) Listens effectively	□ Elicits patient’s reason for seeking health care	□ Manages patient expectations in a manner that minimizes potential for stress, conflict, and misunderstanding. Effectively communicates with vulnerable populations, (at risk patients and families)
23	**Team management (ICS2)**	□ Did not achieve level 1	□ Recognizes other members of the patient care team during case (nurse, techs)	□ Communicates pertinent information to other healthcare colleagues	□ Communicates a clear, succinct, and appropriate handoff with specialists and other colleagues Communicates effectively with ancillary staff

## INSTRUCTOR MATERIALS

**Case Title:** Adolescent with Diabetic Ketoacidosis, Hypothermia and Pneumomediastinum

**Case Description & Diagnosis (short synopsis):** A 12-year-old male presents minimally responsive and hypothermic. On initial evaluation, the patient is found to have a blood glucose reading “high,” but despite initial resuscitation efforts, he becomes more hypoxic requiring intubation. Depending on the ventilator settings chosen, he either improves or deteriorates further into cardiac arrest. Lab and radiology testing show findings consistent with diabetic ketoacidosis (DKA) and associated pneumomediastinum, presumably from the vomiting associated with DKA. The patient requires treatment in 3 phases: 1) recognition and treatment of DKA, 2) intubation with appropriate ventilator settings for a patient with DKA, 3) appropriate disposition.

**Equipment or Props Needed:**

- Adolescent simulator (age of patient can be changed to fit whatever simulator is available)
- IV pole
- IV Normal Saline or Lactated Ringers bag and tubing
- Airway Equipment: nasal cannula, nonrebreather, Glidescope, Mac/Miller Blade, ET Tube, intubation stylet, ETCO2 colorimeter
- Crash cart with associated medications such as epinephrine, sodium bicarbonate, calcium chloride/gluconate
- Glucometer

**Actors needed:**

A nurse, a respiratory therapist (the nurse could also cover this role), a guardian, pediatric intensivist (over the phone)

**Stimulus Inventory:** [Table t3-jetem-8-4-s1]

**Background and brief information:** An obtunded 12-year-old male presents to the emergency department (ED) via private vehicle. Grandmother provides history that the patient had been feeling anxious earlier in the day with numerous bouts of vomiting. The patient has no previous medical problems and is on no medications.

**Initial presentation:** Presents obtunded via private vehicle

**How the scene unfolds:** There are 3 stages to this simulation:

Stage 1) Patient will be mildly hypoxic, hypothermic, and tachycardic and will require supplemental oxygen and intravenous (IV) fluid resuscitation with consideration for warming. He will be found to be in DKA and will receive appropriate treatment with IV fluids and insulin.
Stage 2) Patient will continue to become more hypoxic requiring intubation. Learners will need to get a chest x-ray to articulate appropriate ventilator settings.
Stage 3) If appropriate ventilator settings are chosen (specifically respiratory rate), the patient will stabilize. If inappropriate ventilator settings are chosen, the patient will deteriorate to asystole requiring one round of ACLS and correction of vent settings. In our simulation, two of our groups did not put in appropriate ventilator settings and the patient went into asystole. One group quickly recognized their error. The third group required prompting from the nurse who asked, “What are the patient’s ventilator settings?” The patient will then be dispositioned to the Pediatric Intensive Care Unit (PICU) after discussion with the admitting team. The family will be updated throughout the case. In our simulation, senior learners were paired with junior learners. However, in the setting of a case where there are only junior learners, ventilator settings may be provided, and cardiac arrest omitted.

**Critical actions:**

**Stage 1 Actions**

1. Obtain history from grandparent
2. Obtain Vital Signs including temperature
3. Consider Warming
4. Check labs including blood sugar
5. Treat hyperglycemia with IV fluids and insulin

**Stage 2 Actions**

1. Intubate patient
2. Input correct ventilator settings

**Stage 3 Actions**

1. If patient undergoes cardiac arrest from improper ventilator settings, recognize error and discuss need for continuing increased respiratory rate after intubation
2. Order x-ray chest
3. Recognize pneumomediastinum
4. Admit to PICU

**Case Title:** Adolescent with Diabetic Ketoacidosis, Hypothermia and Pneumomediastinum

**Chief Complaint:** Unresponsive[Table t4-jetem-8-4-s1]

**General Appearance:** Obtunded with Kussmaul breathing

**Primary Survey:**

- **Airway:** Obtunded with Kussmaul breathing
- **Breathing:** Kussmaul breathing with clear breath sounds bilaterally
- **Circulation:** Central and peripheral pulses present, tachycardic

**History:**

- **History of present illness:** The patient was feeling unwell for three days with nausea, vomiting, and fatigue. He seemed anxious and short of breath this morning; his stepmother thought this may be a panic attack and gave 0.5 mg orally of lorazepam. The patient has no history of anxiety, and the medication belonged to the stepmother.
- **Past medical history:** None
- **Past surgical history:** None
- **Patient’s medications:** None
- **Allergies:** None
- **Social history:** No tobacco, alcohol, or drugs, lives at home with parents, not sexually active
- **Family history:** None

**Secondary Survey/Physical Examination:**

- **General appearance:** Obtunded and unresponsive; Glasgow Coma Score (GCS) 8: Mumbling Sounds (2) Does not open eyes (1) Localizes Pain (5)
- **HEENT:**
  ○ **Head:** within normal limits (wnl)
  ○ **Eyes:** Pupils 4mm reactive bilaterally
  ○ **Ears:** wnl
  ○ **Nose:** wnl
  ○ **Throat:** wnl
- **Neck:** wnl
- **Heart:** Tachycardic with no murmurs
- **Lungs:** Kussmaul breaths, clear bilateral breath sounds
- **Abdominal/GI:** wnl
- **Genitourinary:** wnl
- **Rectal:** wnl
- **Extremities:** wnl
- **Neuro:** Glasgow Coma Scale 8; no eye opening, flexion to pain, incomprehensible sounds
- **Skin:** Cold
- **Lymph:** wnl
- **Psych:** Unable to assess[Table t5-jetem-8-4-s1]

*Chest radiograph*

Image Source: Author’s Own Image[Fig f1-jetem-8-4-s1]

*Electrocardiogram*

Image Source: Author’s Own Image[Fig f2-jetem-8-4-s1][Table t6-jetem-8-4-s1][Table t7-jetem-8-4-s1][Table t8-jetem-8-4-s1][Table t9-jetem-8-4-s1]

## OPERATOR MATERIALS

[Table t10-jetem-8-4-s1]

**Diagnosis:**

Diabetic ketoacidosis with hypothermia and pneumomediastinum

**Disposition:**

Pediatric Intensive Care Unit

## DEBRIEFING AND EVALUATION PEARLS

### Adolescent with Diabetic Ketoacidosis, Hypothermia and Pneumomediastinum

#### Pearls: Diabetic Ketoacidosis

- **Background:** Diabetic Ketoacidosis (DKA) is a life-threatening illness that must be identified quickly. It is often the first presentation of Type 1 diabetes in the pediatric population. Common symptoms include nausea, vomiting, fatigue, abdominal pain, altered mental status. DKA leads to profound acidemia, and without treatment can deteriorate to cardiovascular collapse.
- **Etiology:** Incidence of Type 1 diabetes is 22.3 per 100,000 patients per year with 1/3 of those patients presenting in the setting of diabetic ketoacidosis.^3^ Risk factors for diabetic ketoacidosis include low socioeconomic status, non-white patients, and poor access to healthcare. Inciting factors for DKA include illness, new medication, or recreational drug/alcohol use.
- **Diagnostic testing:** Initial finger stick blood glucose of >250 mg/dL should warrant investigation for DKA. Basal metabolic panel (BMP) should be ordered to evaluate potassium and anion gap; arterial or venous blood gas will assess level of acidemia in the patient, and a beta-hydroxybutyrate (or other measure of acetone) can be considered as adjunctive testing. Imaging and lab testing to evaluate for inciting causes of DKA should also be considered.
- **Treatment options:** Treatment of diabetic ketoacidosis in the pediatric population is performed in a multi-step fashion. The first treatment is usually IV fluids with a 10–20ml/kg fluid bolus. More aggressive hydration initially is contraindicated because there is concern for cerebral edema in pediatric patients who are given too much volume in the setting of DKA. After initial fluid bolus, maintenance fluids can be started at 1.5 × maintenance rate; however, fluids should be monitored closely and altered based on patient’s glucose, anion gap, and potassium. In the setting of hypokalemia, potassium supplementation is started with fluid resuscitation, and no insulin is started until the potassium has corrected. If the initial potassium level is normal, insulin and potassium are started, and if the initial potassium is high, fluids alone are started.^4^ Insulin should be started at between .05–.1unit/kg/hr. Potassium levels, glucose levels, and anion gap will be drawn frequently to monitor progress. Sodium bicarbonate should be avoided unless there are signs of severe respiratory or circulatory failure.

#### Pearls: Hypothermia

- Must evaluate degree of hypothermia based on severity and core body temperature. If body temperature is greater than 32°C, then management can focus on removal of wet clothes and placing in a warm environment with warm blankets. For body temperatures below that but in patients who are still hemodynamically stable, options include a forced air warming blanket. If there is any sign of hemodynamic instability in patients with hypothermia, treatment should move towards warm IV fluids, intrapleural, or intraperitoneal warm fluid wash. ECMO may be indicated in extreme cases. The recommended rate of rewarming is .5–2°C per hour.^5^

#### Pearls: Ventilator Management

- While patients in DKA can present very obtunded, intubation should only be considered if truly necessary because a patient is able to improve his or her acidemia with an increased respiratory rate that is difficult to match using a ventilator. Patients with DKA have Kussmaul breathing due to respiratory compensation from severe metabolic acidosis. Normal ventilator settings will worsen acidemia and increase mortality.^2^ However, given a DKA patient’s higher risk for acute respiratory distress syndrome (ARDS), peri-intubation lung protective tidal volumes should be maintained.^6^ This means primary focus should be on maintaining a tidal volume similar to the patient’s volumes prior to intubation. Diabetic ketoacidosis is associated with decreased total body stores of magnesium and potassium which in turn causes patients to have concerning muscle weakness which can prolong patient’s ventilator time.^8^

#### Pearls: Pneumomediastinum

- Pneumomediastinum is a rare condition of free air in the mediastinum. Generally seen in young, thin males, it is commonly preceded by a history of nausea with vomiting, smoking, trauma, or recent medical instrument use. Treatment is generally supportive by providing antiemetics, analgesia, bedrest, and supplemental oxygen as needed. For patients with extensive subcutaneous emphysema or hemodynamic instability, thoracic surgery can be involved, and these patients can sometimes be treated in the operating room. Prophylactic antibiotics are generally not indicated.^7^

#### Pearls: Cerebral Edema

- Cerebral edema must be on the differential diagnosis for patients with DKA and altered mental status. Signs of cerebral edema include worsening vomiting, mental status change, hypertension, bradycardia, or worsening headache. This has found to be associated with bicarbonate use and is sometimes thought to be associated with more aggressive IV fluid rates; however, this is controversial. Should the provider have concerns for cerebral edema, head of bed should be elevated and urgent consult to PICU should be placed.^8^

## SIMULATION ASSESSMENT

*Adolescent with Diabetic Ketoacidosis, Hypothermia and Pneumomediastinum*

Learner: _________________________________________

***Assessment Timeline***

This timeline is to help observers assess their learners. It allows observer to make notes on when learners performed various tasks, which can help guide debriefing discussion.[Table t11-jetem-8-4-s1]

**Critical Actions:**

**Stage 1 Actions:**

□ Vital signs
□ Obtain history from grandparent
□ Discuss warming
□ Check blood sugar
□ Administer IV fluids

**Stage 2 Actions**

□ Intubate patient
□ Input correct ventilator settings
□ If patient undergoes cardiac arrest from improper ventilator settings, discuss cause
□ Start insulin and discuss IV fluids based on lab results
□ Recognize pneumomediastinum
□ Admit to PICU

**Summative and formative comments:**

**Milestones assessment:** [Table t12-jetem-8-4-s1]
