# Supplementary material for: Adolescent with Diabetic Ketoacidosis, Hypothermia and Pneumomediastinum
Source: J Educ Teach Emerg Med. 2023 Oct 31;8(4):S1–S24. doi: 10.21980/J8FP8J (PMC10631814; doi:10.21980/J8FP8J)
Supplement: Supplementary file 1 [file jetem-8-4-S1-supp1.pptx]

## Slide 1
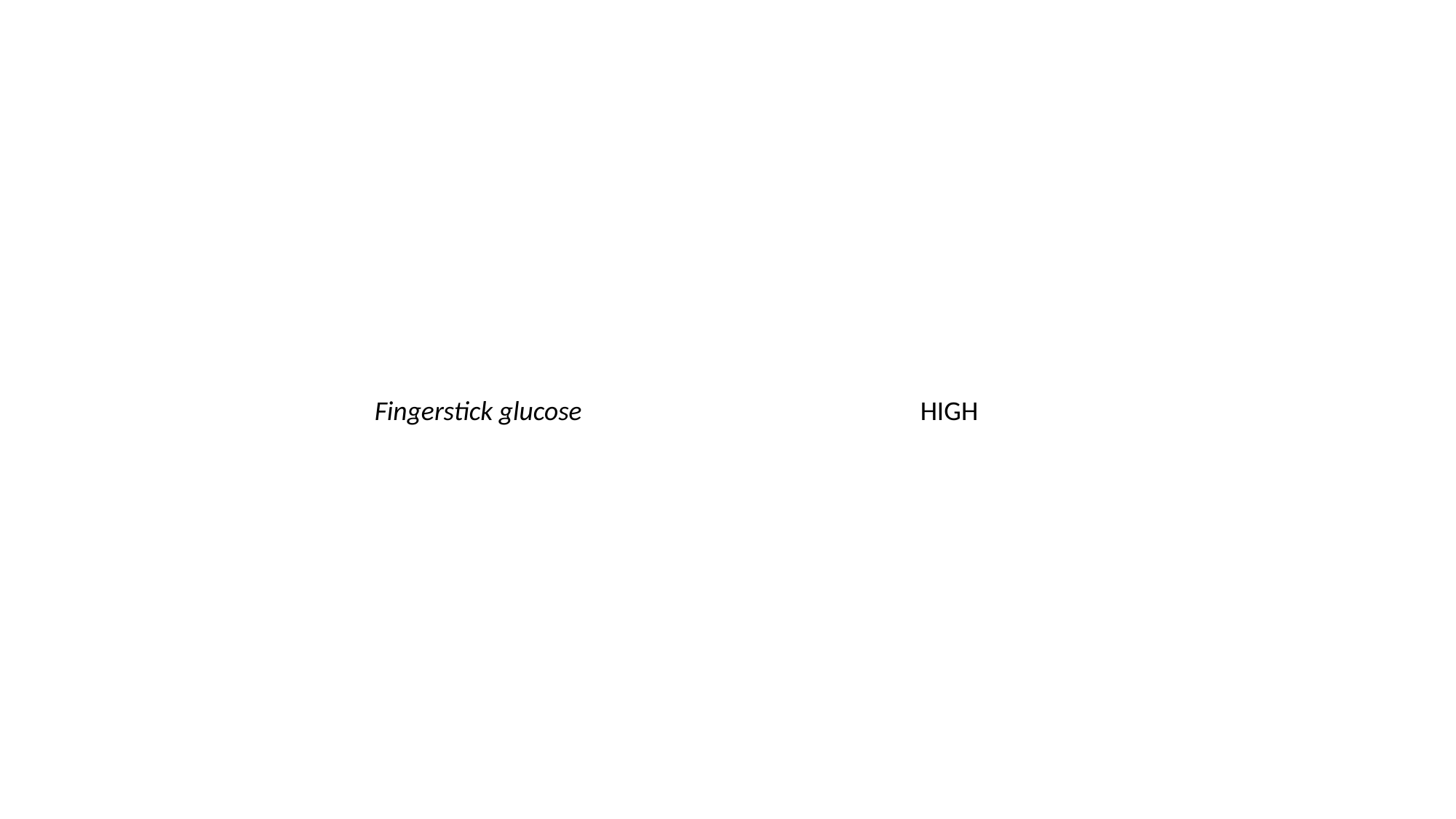

Fingerstick glucose				HIGH

## Slide 2
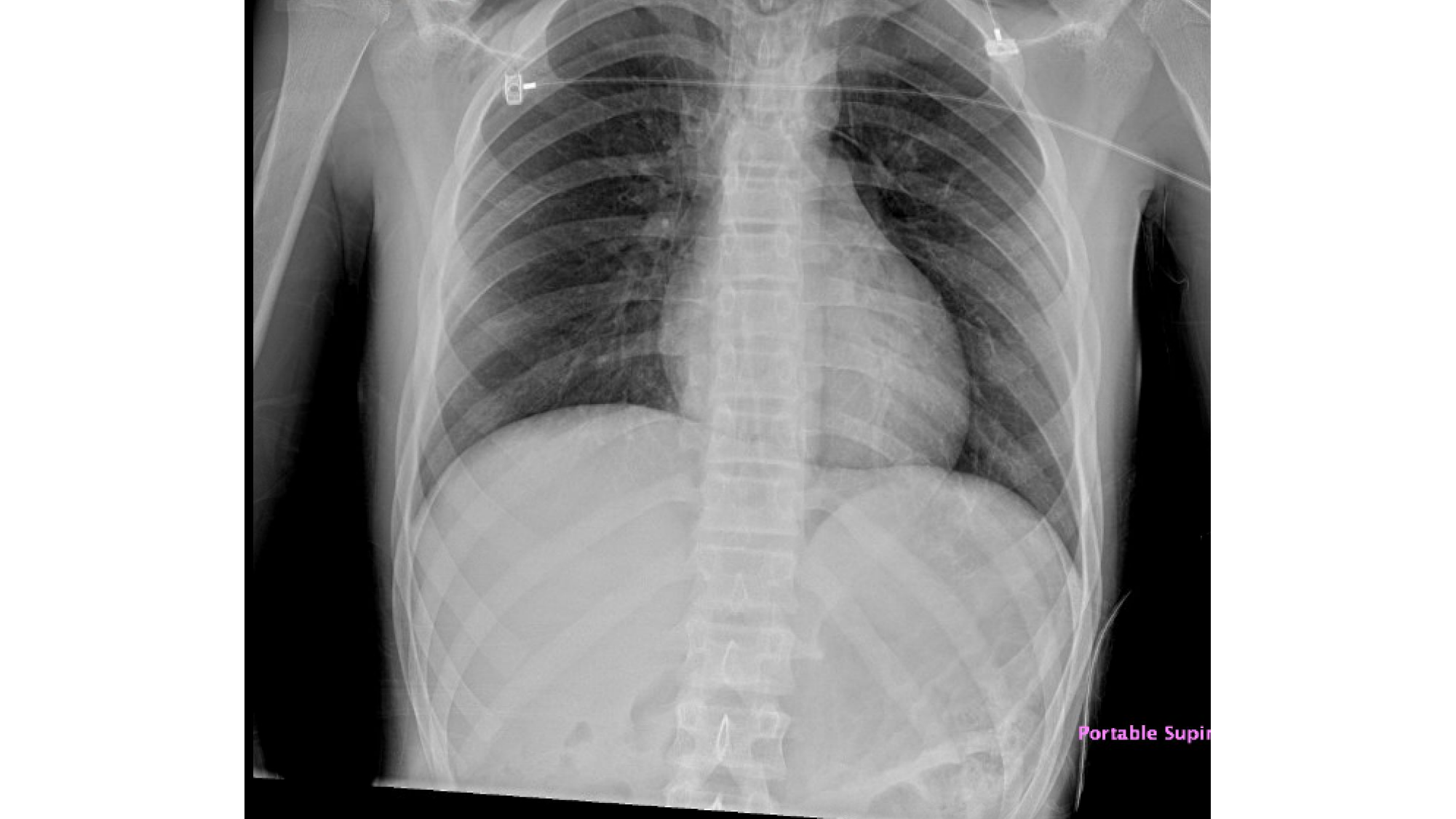

## Slide 3
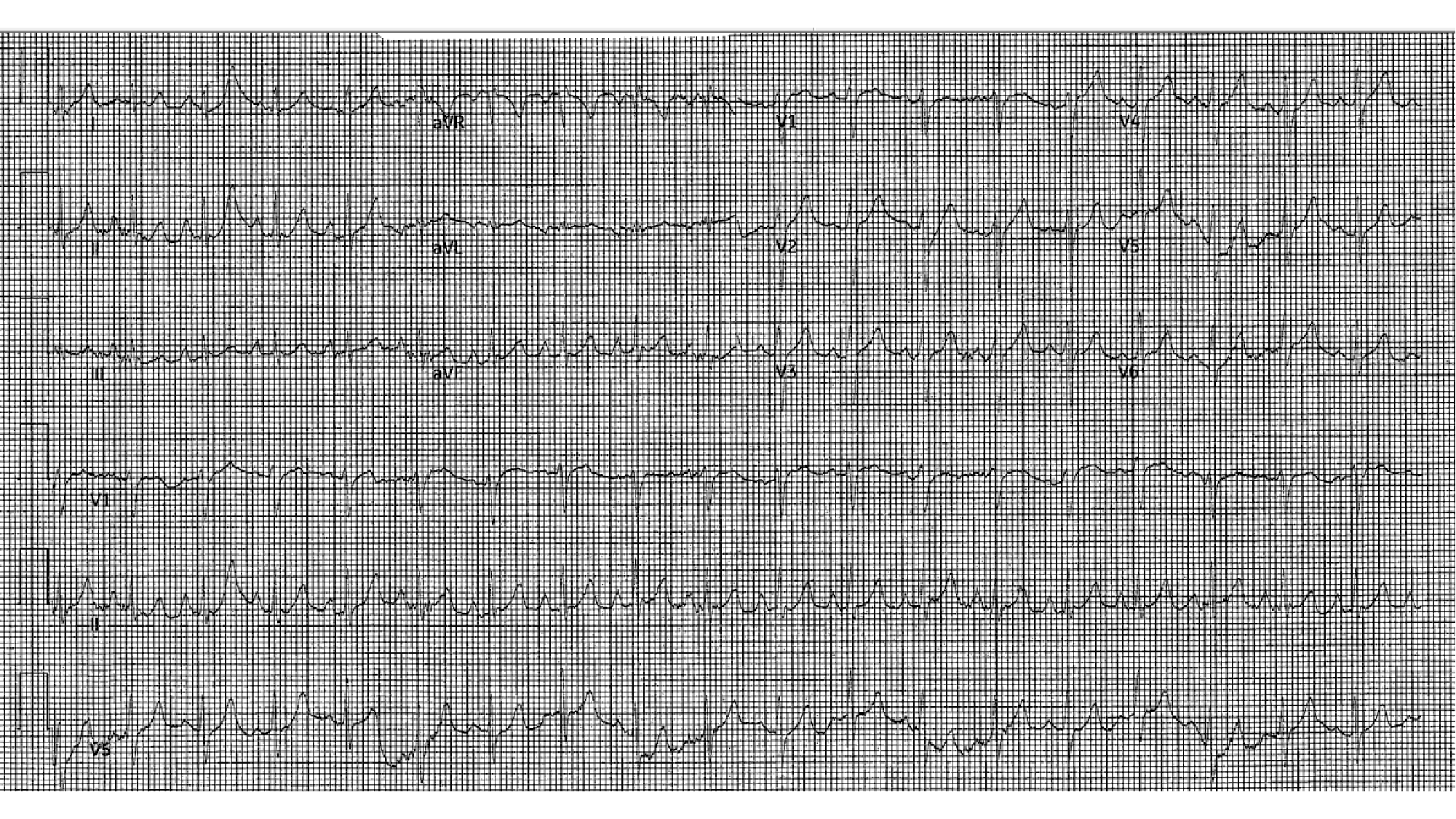

## Slide 4
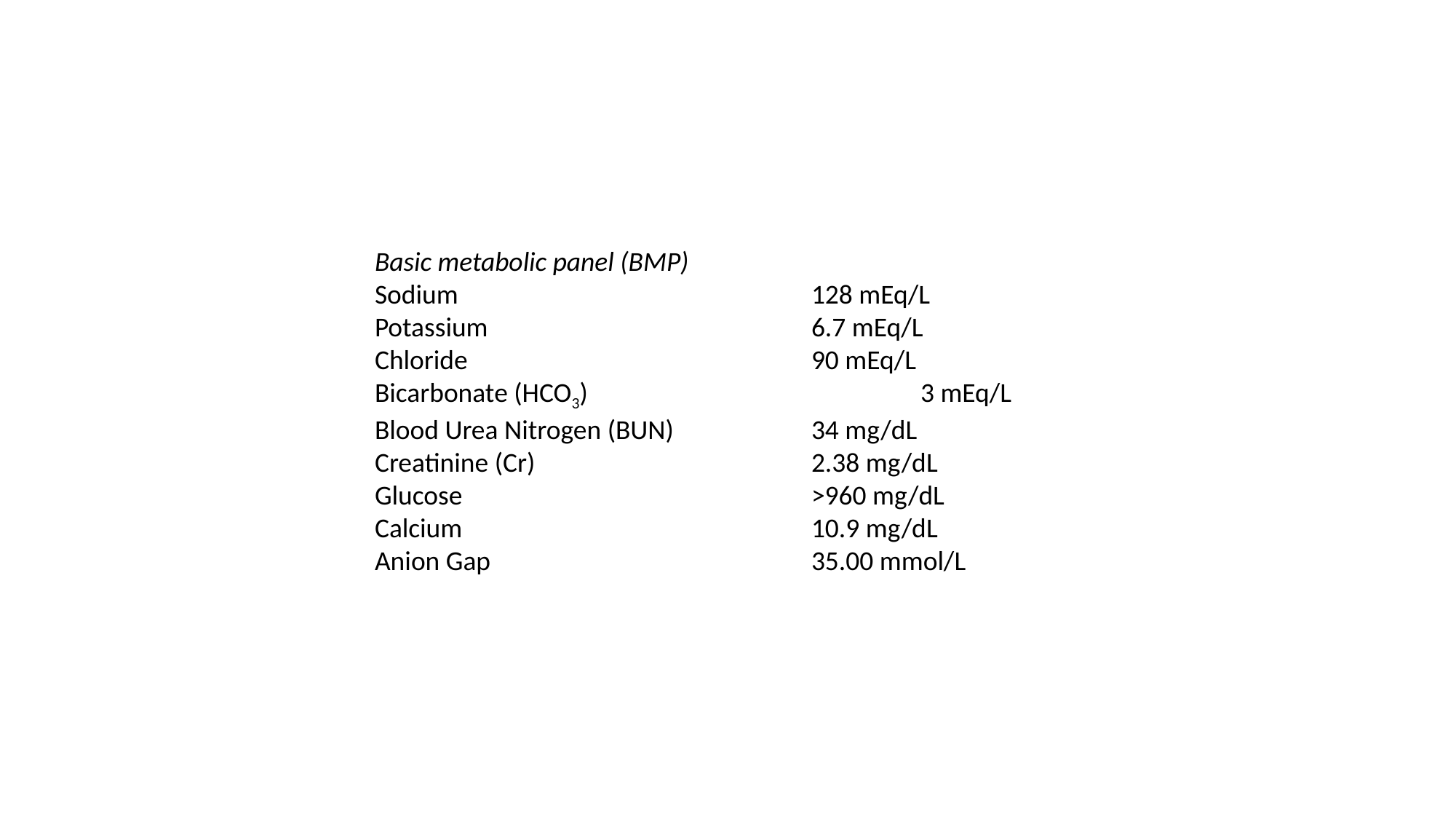

Basic metabolic panel (BMP)
Sodium				128 mEq/L
Potassium				6.7 mEq/L
Chloride				90 mEq/L
Bicarbonate (HCO3)				3 mEq/L
Blood Urea Nitrogen (BUN)		34 mg/dL
Creatinine (Cr)				2.38 mg/dL
Glucose				>960 mg/dL
Calcium				10.9 mg/dL
Anion Gap				35.00 mmol/L

## Slide 5
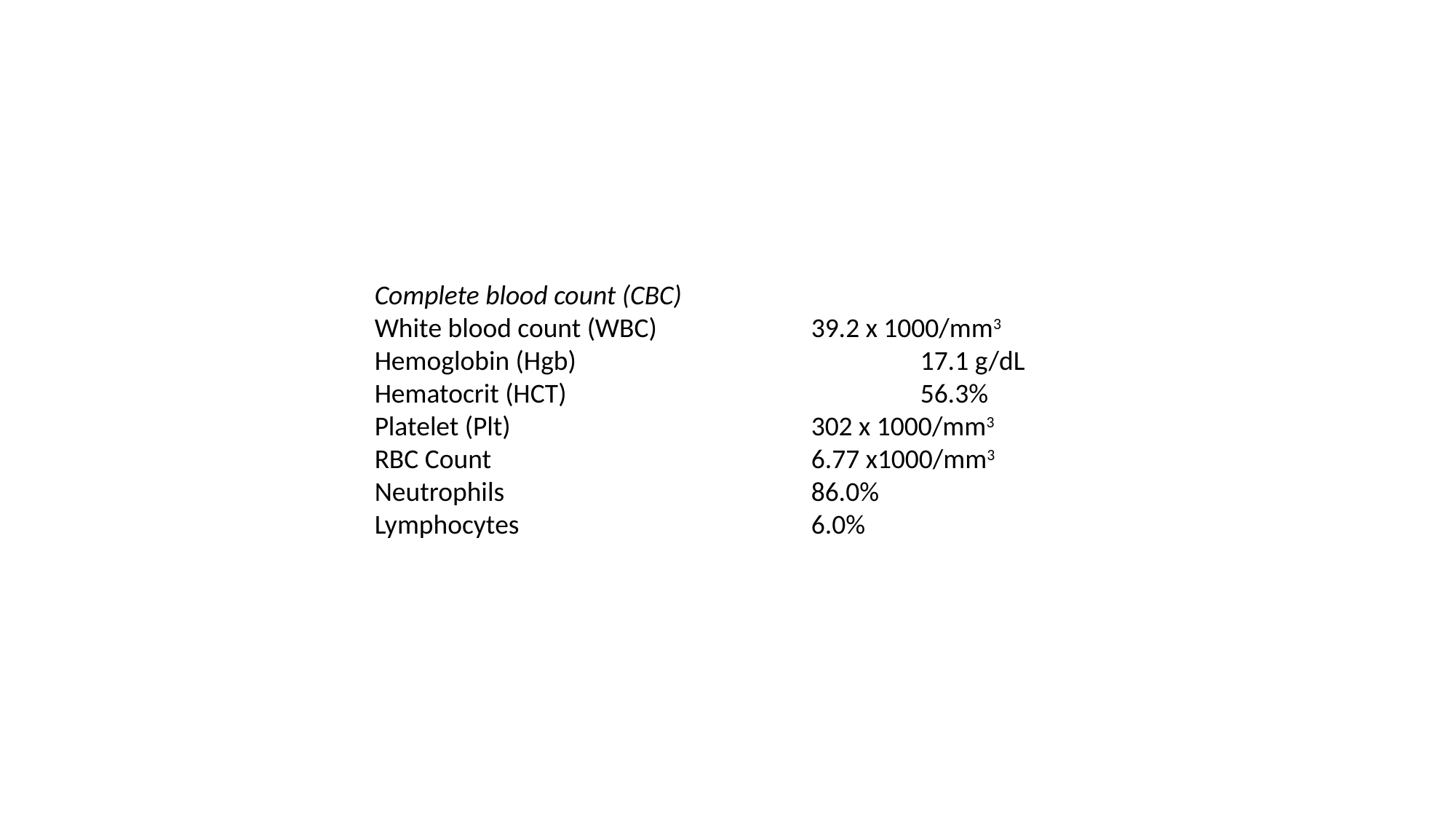

Complete blood count (CBC)
White blood count (WBC)		39.2 x 1000/mm3
Hemoglobin (Hgb)				17.1 g/dL
Hematocrit (HCT)				56.3%
Platelet (Plt)				302 x 1000/mm3
RBC Count				6.77 x1000/mm3
Neutrophils				86.0%
Lymphocytes				6.0%

## Slide 6
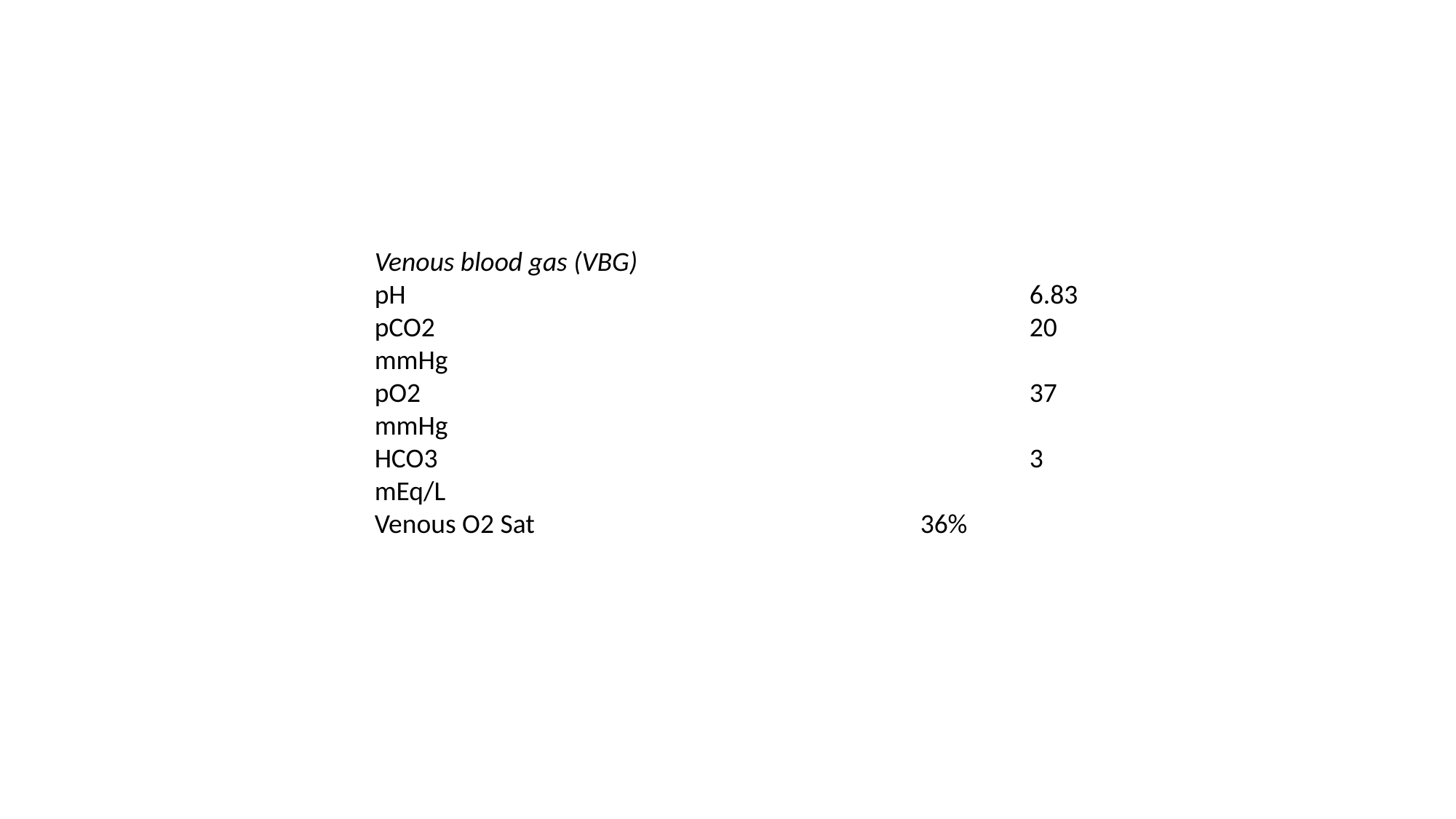

Venous blood gas (VBG)
pH						6.83
pCO2						20 mmHg
pO2						37 mmHg
HCO3						3 mEq/L
Venous O2 Sat				36%

## Slide 7
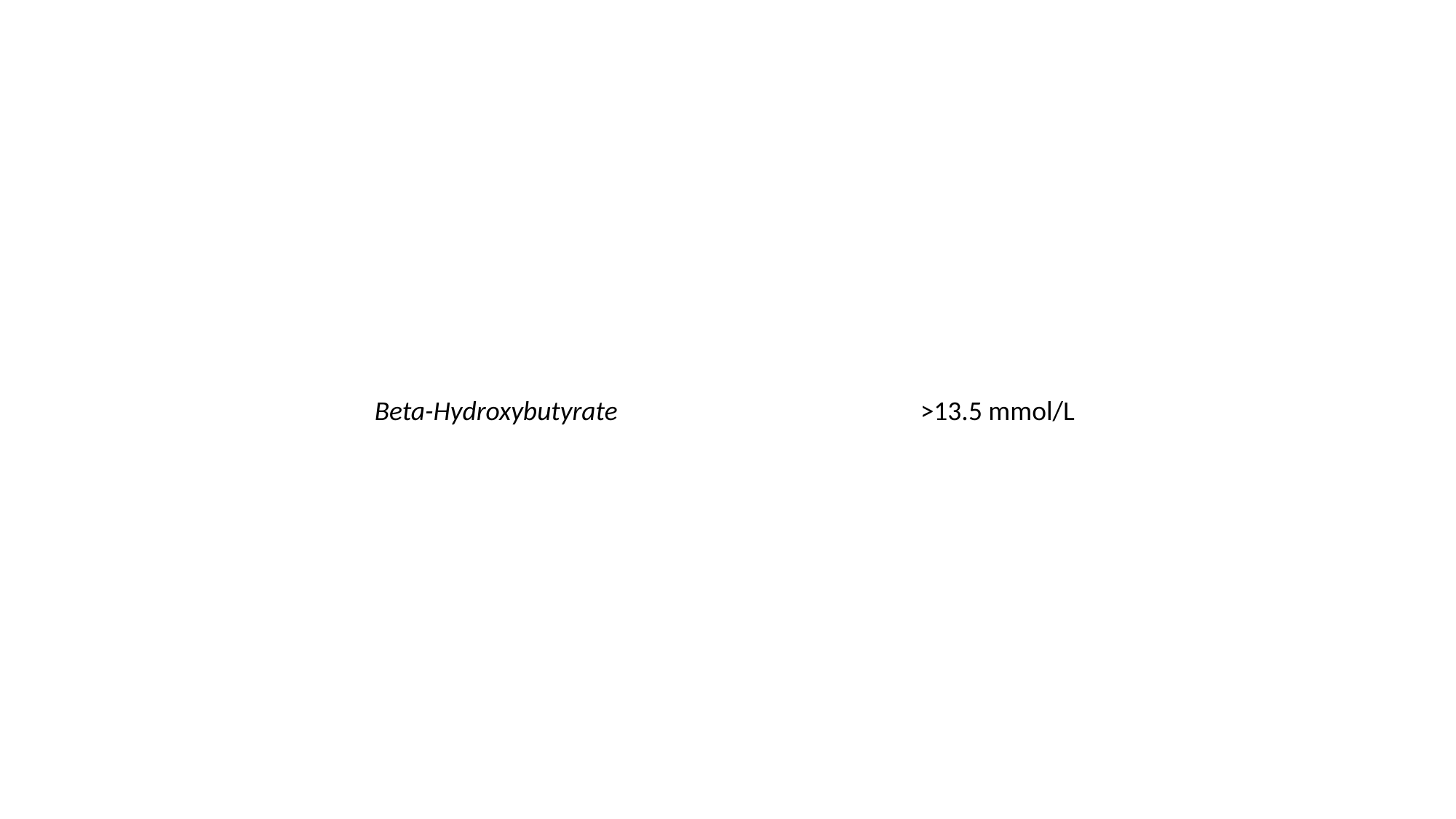

Beta-Hydroxybutyrate			>13.5 mmol/L
